# *Pilea umbrosa* ameliorate CCl_4_ induced hepatic injuries by regulating endoplasmic reticulum stress, pro-inflammatory and fibrosis genes in rat

**DOI:** 10.1186/s12199-020-00893-2

**Published:** 2020-09-11

**Authors:** Irum Naz, Muhammad Rashid Khan, Jawaid Ahmed Zai, Riffat Batool, Zartash Zahra, Aemin Tahir

**Affiliations:** Faculty of Biological Sciences, Department of Biochemistry, Quaid-i-Azam University, Islamabad, Pakistan

**Keywords:** Liver, Antioxidant, ER stress, Cytokines, RT-PCR

## Abstract

**Background:**

*Pilea umbrosa* (Urticaceae) is used by local communities (district Abbotabad) for liver disorders, as anticancer, in rheumatism and in skin disorders.

**Methods:**

Methanol extract of *P. umbrosa* (PUM) was investigated for the presence of polyphenolic constituents by HPLC-DAD analysis. PUM (150 mg/kg and 300 mg/kg) was administered on alternate days for eight weeks in rats exposed with carbon tetrachloride (CCl_4_). Serum analysis was performed for liver function tests while in liver tissues level of antioxidant enzymes and biochemical markers were also studied. In addition, semi quantitative estimation of antioxidant genes, endoplasmic reticulum (ER) induced stress markers, pro-inflammatory cytokines and fibrosis related genes were carried out on liver tissues by RT-PCR analysis. Liver tissues were also studied for histopathological injuries.

**Results:**

Level of antioxidant enzymes such as catalase (CAT), superoxide dismutase (SOD), peroxidase (POD) and glutathione (GSH) decreased (*p* < 0.05) whereas level of thiobarbituric acid reactive substance (TBARS), H_2_O_2_ and nitrite increased in liver tissues of CCl_4_ treated rat. Likewise increase in the level of serum markers; alanine transaminase (ALT), aspartate transaminase (AST), alkaline phosphatase (ALP) and total bilirubin was observed. Moreover, CCl_4_ caused many fold increase in expression of ER stress markers; glucose regulated protein (GRP-78), x-box binding protein1-total (XBP-1 t), x-box binding protein1-unspliced (XBP-1 u) and x-box binding protein1-spliced (XBP-1 s). The level of inflammatory mediators such as tumor necrosis factor-α (TNF-α), interleukin-6 (IL-6) and monocyte chemoattractant protein-1 (MCP-1) was aggregated whereas suppressed the level of antioxidant enzymes; γ-glutamylcysteine ligase (GCLC), protein disulfide isomerase (PDI) and nuclear erythroid 2 p45-related factor 2 (Nrf-2). Additionally, level of fibrosis markers; transforming growth factor-β (TGF-β), Smad-3 and collagen type 1 (Col1-α) increased with CCl_4_ induced liver toxicity. Histopathological scrutiny depicted damaged liver cells, neutrophils infiltration and dilated sinusoids in CCl_4_ intoxicated rats. PUM was enriched with rutin, catechin, caffeic acid and apigenin as evidenced by HPLC analysis. Simultaneous administration of PUM and CCl_4_ in rats retrieved the normal expression of these markers and prevented hepatic injuries.

**Conclusion:**

Collectively these results suggest that PUM constituted of strong antioxidant chemicals and could be a potential therapeutic agent for stress related liver disorders.

## Background

Hepatic fibrosis is a pathological condition characterized by activation of hepatic stellate cells (HSCs), extracellular matrix (ECM) proteins accumulation and formation of pseudo lobules or nodules [1]. Due to its frequent recurrence, it affects the people all around the world and causes up to one million deaths in almost 184 countries [2, 3]. The clinical manifestation associated with chronic liver disease includes inflammation, infection, cirrhosis and metabolic disorders which arise as a consequence of exogenous chemicals, drugs and chemotherapeutic agents resulting in over production of reactive oxygen species (ROS). Therefore, if hepatic fibrosis left untreated at earlier stages, it can lead to irreversible cirrhosis or fulminant hepatic carcinoma [4–6]. Carbon tetrachloride (CCl_4_) has been extensively used in animal models to elucidate the mechanism of hepatic injury and for analyzing biomarkers discovery. It stimulates cytochrome P-450 enzyme (CYP) system in endoplasmic reticulum (ER) to metabolize into trichloromethyl radicals (•CCl_3_) and trichloromethyl peroxy radical (•CCl_3_O_2_). These radicals inaugurate lipid peroxidation and compel oxidative stress that ultimately causes liver cell damage. Furthermore, CCl_4_-induced liver toxicity is accompanied by elevation of inflammatory cytokines such as interleukin (IL-1β), IL-6, tumor necrosis factor (TNF-α), and chemokines e.g. monocyte chemo attractant protein-1 (MCP-1) which causes severe hepatocyte inflammation [7, 8].

Under transient conditions, both ROS and antioxidant defense systems work in an equal proportion. On the other hand, the chronic stress which causes augmentation of ROS in subcellular organelles, as in ER lumen, disturbs the process of protein folding and causes aggregation of unfolded or misfolded proteins, is referred as ER stress. In order to decrease the stress, cells eventually deploy the unfolded protein response (UPR) by restricting aggregation of unfolded protein over transient stress exposition. Activated UPR causes up-regulation of intra-ER chaperones mainly the glucose-regulated protein (GRP-78) associated with protein folding. It is released from ER and provokes three sensory cascades: inositol-requiring protein 1 alpha (IRE1-α), protein kinase RNA-like ER-associated kinase (PERK), and activating transcription factor (ATF-6). These sensory cascades re-establish homeostasis synergistically by promoting transcription of genes for protein folding and terminating general translation [8]. However, if underlying mechanism fails in adaptation of the cells towards ER stress, UPR leads to apoptosis of susceptible cells. Prolonged activation of UPR stimulates IRE1-α branch endoribonuclease activity which triggers cleavage of x box-binding protein-1 (XBP-1) mRNA to generate an effective transcription factor x-box binding protein1-spliced (XBP-1 s). It subsequently transcribes other proteins and causes ER-associated degradation thereby reducing ER stress [9]. Therefore, some recent reports suggested that significantly increased transcription level of GRP-78, XBP-1u and XBP-1 s can be considered as ER stress markers [10–12].

In response to liver fibrosis, activated HSCs and Kupffer cells participate in synthesis of ECMs such as collagen type-1 [13]. Alongside, damage cells release inflammatory cytokines and growth factors, which are involved in promoting HSCs and eventually aggravate liver fibrosis. Reports indicated that inflammatory cells and fibrosis activates the transforming growth factor-β (TGF-β)/ Smad signaling pathway. Upon binding of TGF-β with its transmembrane serine/threonine kinase type-I receptor (TβRI), Smad2 and Smad3 get phosphorylated which in turn activates Smad4. This complex is recruited towards the nucleus and binds with TGF-β-responsive genes promoter to encode genes for collagen type-1 and other ECM proteins. Therefore, evidence suggested that TGF-β1/Smad signaling is a potential target to prevent HSCs activation, which subsequently inhibits ECM synthesis and ameliorates hepatic fibrosis. Other factors which diminish ER stress are implicated in reducing hepatic fibrosis involve chaperones such as 4-PBA [14] and has been declared as anti-fibrotic agent, under the influence of UPR. Nevertheless, fibrosis triggered by the complex mechanism of UPR requires a deeper understanding of the mechanism that plays a role in activating HSCs that can contribute to evade fibrosis.

Compounds with antioxidant aptitude related to the prevention and treatment of oxidative damage induced by intracellular ROS level have been studied. Various taxa within the Urticaceae family have been explored for its medical potential against a wide array of diseases including cardiovascular, inflammatory, metabolic and respiratory disorders [15–17]. Among them some well-known plants such as *Elatostema umbellatum*, *Pilea microphylla* and *Urtica dioica* owing phenolic contents have been documented with antioxidant properties [18]. *Pilea umbrosa* Blume commonly known as Sikri booti is a perennial herb. It is 30–50 cm in height often covered with hairs. It is found at an altitude of 1200–2500 m in Himalayan forest of Pakistan*.* The plant has been reported for its therapeutic use in liver disorders, as anticancer and in rheumatism [19]. The plant is also used to cure skin diseases by traditional people. The current study has been disbursed to assess the protective potential of methanol extract of *P. umbrosa* on CCl_4_ induced hepatotoxicity in Sprague Dawley male rats. Therefore, the protecting proceeding and underlying mechanisms were evaluated, to assess the rationale of *P. umbrosa* in treating hepatic disorders and suggesting its clinical use.

## Materials and methods

### Plant material collection and identification

Fresh plant of *Pilea umbrosa* was collected from mountain top of Mushkpuri, Abbottabad district (7550 ft. altitude) of Pakistan in October 2017. The plant sample was recognized and validated by Dr. Syed Aneel Ahmad Gilani at Pakistan Museum of Natural History, Islamabad. The voucher specimen (#129917) was submitted at Herbarium of Plant Sciences, Quaid-i-Azam University, Islamabad, Pakistan.

### Extraction and isolation

The bulk of *P. umbrosa* plant was made free of dirt and dried for 9 days. Thereafter, it was crushed by Willy mill into fine powder (1 kg) of 80 mesh size. Methanol extract (PUM) using standard ratio of 1:4 was obtained twice by immersing in 95% of methanol for a week. The material was repeatedly shaken and mixed until all the constituents get dissolved in solvent. It is then filtered and concentrated under vacuum with rotary evaporator at (25 °C ± 5) to get PUM (50 g) and stored at 4 °C.

### HPLC-DAD analysis of PUM

HPLC-DAD analysis of PUM was carried out with the help of Agilent technology-1200 series, Germany, equipped with UV detector and reverse phase analytical column Zorbex plus RSC8 (Agilent U.S.A); particle size 5 μm; capacity 25 ml for separation. The mobile phase composed entirely of elution solvent A as acetonitrile (5%); methanol (10%); water (85%); acetic acid (1%) and elution solvent B as acetonitrile (40%); methanol (60%); acetic acid (1%) following gradient program [20]. The flow rate was maintained at 1 ml/min keeping injection volume at 1 ml. Prior to analysis, samples solutions (10 mg/ml) were diluted by methanol (HPLC grade > 99.9%) (Sigma Aldrich) and filtered via 0.45 μm membrane filters. The column was renovated after every turn for 10 min. The standards; rutin and gallic acid were eluted at 257 nm, catechin at 279 nm, caffeic acid and apigenin at 325 nm, myricetin, quercetin and kaempferol eluted at 368 nm, respectively. The compounds were analyzed in triplicates and quantified at different wavelength using standard’s peaks.

### Ethical statement

Animal studies were performed at primate facility after ethical approval (Bch# 0323) by review board of ethics committee of Quaid-i-Azam University, Islamabad, Pakistan. The scientific animal experimentation was performed according to the standard guidelines of National Institute of Health, Islamabad, Pakistan.

### Acute toxicity study

In a preliminary study, the acute toxicity study was determined by using the procedure approved by [11]. Female rats (*n* = 5) were given 50 mg/kg body weight (bw) dose of PUM and were investigated for any deterioration and weight loss and no signs of distress were seen. Afterward, the procedure was aspired at different concentrations (100, 500, 1000, 1500 and 3000 mg/kg) of PUM to 5 female rats (for each respective dose) and left for 3 weeks to observe mortality. Since doses were well tolerated by rats, therefore one tenth (300 mg/kg bw) and one twentieth (150 mg/kg bw) of the highest dose was chosen for the appraisal of hepatoprotective effect of PUM. At the end of procedure, the rats administered with 1500 mg/kg and 3000 mg/kg were dissected in order to collect blood via cardiac puncture for hematological parameters.

### Animal treatment

Forty-two male Sprague-Dawley rats (weighing 125 ± 5 g) acquired from primate facility of Quaid-i-Azam University were employed for experimentation. The rats were kept at ambient temperature (22 ± 2 °C) under 12 h-dark and l2 h-light cycles, relative humidity of (50% ± 5%) and retained under pathogen free condition to verify the absence of exclusion criteria. Rats had acquired access to standard rodent diet and at liberty to water, unless otherwise noted.

#### Experimental design

Before commencement of experimentation, 42 rats were divided into seven groups (six rats in each). The procedure was carried out on alternate days for 8 weeks (24 doses). Group I rats served as a control, received 1 ml/kg bw of 0.9% saline along with regular chow diet and kept under standard conditions. Next cohort of rats (Group II) was injected only with intra-peritoneal treatment of 1 ml/kg bw of CCl_4_ (Sigma-Aldrich) diluted with olive oil (CCl_4_: olive oil; 3:7 v/v) thrice a week on alternate days, to induce hepatotoxicity. Group III was treated with silymarin as a standard drug at 200 mg/kg bw concentration after 24 h of CCl_4_ intra-peritoneal injection to rat. Group IV and V were administered with (150 mg/kg bw and 300 mg/kg bw) PUM by gavage after 24 h of intra-peritoneal injection of CCl_4_ to rat. Whereas, Group VI and VII were treated alone with PUM extract at 150 mg/kg and 300 mg/kg bw dose, respectively on alternate days of 2 weeks. Each rat was observed for any visible sign of deterioration at different time points from dose administration. Following experiment completion, the treatment was discontinued for 3 days (substituted for standard chow). Thereafter, the rats were euthanized and sacrificed; their livers were removed, weighed and sectioned into two pieces. The first section was immediately snapped frozen at − 40 **°**C for molecular and enzymatic analysis, while other section was fixed with 5% formalin for histological examination. Total blood sample was collected for serum analysis.

#### Organ weight and body weight analysis

Rats in all the seven groups were precisely weighed at the beginning and after the completion of experimental procedure and percent increase of body weight was determined for each rat separately. Following dissection; liver was excised, washed with 0.9% saline and its absolute weight was determined. Likewise, relative weight of liver was calculated for each rat of individual group as liver weight/body weight × 100.

#### Biochemical parameters in serum

In order to evaluate hepatotoxicity serum was collected by spinning blood samples at low speed (3000×g for 20 min at 4 °C). Thereafter, level of ALT, AST, ALP, albumin and total bilirubin in serum were determined with an AMP Diagnostic kits (Krenngasse 8010 Graz, Austria) by using clinical biochemical analyzer (Beckman, USA), according to the standard protocol.

#### Biochemical parameters of liver

The frozen liver samples were vigorously homogenized in 1 mM EDTA and (100 mM) potassium phosphate buffer (pH = 7.4); it was retained for 20 min at 4 °C before centrifugation at 1500×g for getting supernatant (crude enzymes). Total protein in tissue was estimated following the standard procedure of [21]. Absorbance of reaction mixture was quantified by spectrophotometer at 595 nm. Bovine serum albumin (BSA) standard curve was generated to analyze the total protein from tissue homogenate.

#### Antioxidant enzyme status in liver

The activity of antioxidant enzymes such as CAT, POD, SOD and glutathione (GSH) was determined by standard procedure of [22–25], respectively. Absorbance was recorded against blank (same reagent without enzyme extracts) at respective wavelength for each assay. The amount of enzyme activities was estimated (1 unit/min) as decrease in absorbance value of 0.01/min of enzyme activity and presented as U/mg of protein for CAT, POD, SOD activity and μM GSH/ mg of protein for glutathione assay.

##### Measurement of lipid peroxidation (TBARS) assay

Level of thiobarbituric acid (TBARS) was determined by using the method of [26]. The activity was recorded as nM TBARS/min/mg tissue at 37 °C by applying molar extinction coefficient (1.56 × 10^5^ M^− 1^ cm^− 1^).

##### Hydrogen peroxide assay (H_2_O_2_)

By the outline of H_2_O_2_–intermediate horseradish peroxidase supported oxidative reaction of phenol red with H_2_O_2_ assay was performed [27]. At the end of reaction, absorbance of supernatant was determined at 610 nm in comparison to blank reagent. H_2_O_2_ amount was recorded as nM H_2_O_2_/min/mg tissue, whereas oxidation of phenol red by H_2_O_2_ was taken as a standard curve.

##### Nitrite assay

For the measurement of nitric oxide metabolite Griess reagent based [28] methodology was used in this study. Following the reaction procedure, the absorbance of resultant mixture was measured at 540 nm. For defining the concentration of nitrite in tissue samples sodium nitrite curve was used.

### RNA extraction

Total RNA was extracted using TRIzol reagent (Invitrogen) from adherent cells of hepatic tissues, as described else [29]. The resulting pellet was eluted in 50 μl of RNAse free water of molecular grade and preserved at − 40 °C. RNA was quantified by using Nano drop, integrity and purity of RNA was assessed by analyzing the absorption ratios 260/280 (~ 2.0) and 260/230 (2.0–2.2).

#### cDNA synthesis

Equimolar concentrations of RNA were successfully converted to cDNA using 2000 ng of total RNA by using Revert Aid First Strand cDNA Synthesis Kit. The incubation was performed in Thermal Gene Cycler (Biometra). The PCR conditions were adjusted as: 42 °C/60 min, 70 °C/5 min and 4 °C/5 min in Thermal Gene Cycler (Biometra). Besides, controls in the absence of reverse transcriptase and non-template were also employed to ensure fidelity of process. The primer sets of target genes, annealing temperatures and expected product size used in real-time PCR reactions are enlisted in Table 1.

**Table 1:** Primer sequences for real-time PCR

Gene	Primer sequences (5′-3′)		Product size (bp)
GRP78	F R	GCAGTCTCCAGCCTACTTG TCCTTCCCCTTGAGAACCTG	103
PDI	F R	AGAACTCCAGGCGGTGTCT GCCATGGAACTGATGAGAG	150
XBP-1 s	F R	CATGGATTCTGACGCTGTTG CTCTGGGGAAGGACATTTGA	110
XBP-1 t	F R	TGTCACCTCCCCAGAACATC ACAGGGTCCAACTTGTCCAG	103
XBP-1u	F R	TGAAGCGCTGCGGAGGACA AGCTGGAGTTTCTGGTTCT	114
TNF-ɑ	F R	GTCTGTGCCTCAGCCTCTTC GCCATGGAACTGATGAGAG	122
IL-6	F R	GCCTGCAGAGATTCAAGTCA GTATCAGTGGGGGTCAGCAG	140
MCP-1	F R	TGTTCACAGTTGCTGCCTGT CGACTCATTGGGATCATCT	141
TGF-β	F R	GCCTGCAGAGATTCAAGTCA GTATCAGTGGGGGTCAGCAG	109
Smad-3	F R	CACCTCCTGGCTACCTGAGT GTTATTGTGTGCTGGGGACA	118
Col-1α	F R	TGTTCAGCTTTGTGGACCTC GACCCTTAGGCCATTGTGT	114
Nrf-2	F R	TCCAGACAGACACCAGTGGA GAATGTCTCTGCCAAAAGC	122
GCLC	F R	GAGAACATCAGGCTCTTTGC AGATGCACCTCCTTCCTCTG	106
β-actin	F R	TCTACAATGAGCTGCGTGTG ACGTACATGGCTGGGGTGT	134

*GRP-78* Glucose regulated protein 78, *PDI* Protein disulfide isomerase, *XBP-1 s* X-box binding protein-1 spliced, *XBP-1 t* X-box binding protein-1 total, *XBP-1u* X-box binding protein-1 unspliced, *TNF-α* Tumor necrosis factor-alpha, *IL-6* Interleukin-6, *MCP-1* Monocyte chemoattractant protein-1, *TGF-β* Transforming growth factor-beta, *Smad-3* Mothers against decapentaplegic homolog- 3, *Col-****1****α* Collagen-1 alpha, *Nrf-2* Nuclear factor erythroid 2–related factor 2, *GCLC* Glutamate-cysteine ligase catalytic subunit

#### Real-time PCR (RT-qPCR) assays

The mRNA expression analysis was performed with real time Thermal Cycler (iCycler, Bio-Rad) according to the manufacturer’s protocol. Briefly, the reaction mixture was prepared by using 2X Scientific Maxima SYBR Green/ROX qPCR master Mix, 1 nl cDNA and 1 nM primers. Real time PCR cycling parameters were adjusted as: 95 °C for 5 min, followed by 40 cycles of (95 °C/15 s; 55 °C/15 s and 72 °C/60 s) with heat-denaturing PCR products a terminal melt curve analysis was done over 35 °C gradient at 0.2 °C/s from 60 °C to 95 °C. To ensure PCR fidelity melting curve analysis and agarose gel electrophoresis was performed. Expression level of genes was estimated via internal normalization by beta actin as reference gene. Relative quantification of target gene was performed by using the 2^−ΔΔCt^ method. Results were expressed as relative mRNA abundance over values obtained from control animals. The data was assembled with the aid of Microsoft Excel and statistically analyzed using REST software on ΔCts with the formulas: ΔCt = Ct_Mean (target) - Ct_Mean (control), ΔΔCt = ΔCt_treated- ΔCt_control.

### Histopathology analysis

For histological analysis, paraffin embedded 5 μm thin sections of liver tissue were stained with hematoxylin and eosin (H&E). The slides were well examined to analyze the protective aptitude of PUM against CCl_4_ intoxicated liver and results were interpreted using the histological scoring system by adding the scores of steatosis (0–3), leukocyte infiltration (0–3), fatty degeneration (0–3). perivascular and lobular necrosis (0–3), cellular hypertrophy (0–3) and sinusoidal obstruction (0–3). Thorough examination of the slides was performed under compound microscope (DIALUX 20 EB) at 40X magnification and photographed with HDCE-50 B camera. Injuries induced with CCl_4_ in liver of rat and amelioration with extract were graded as; +/−; very minor, +; lower, ++/−; medium, ++; severe and +++; very severe grade.

### Statistical analysis

Results are displayed for continuous variables as the means ± standard deviation on GraphPad Prism v.6.0. One-way analysis of variance (ANOVA) on Statistics 8.1 was performed for verification of differences among groups. For biochemical markers, results were analyzed using Microsoft Excel 2010. Tukey HSD Post hoc comparison among the various treatment groups was carried at *P* < 0.05.

## Results

### HPLC-DAD analysis of PUM

**Fig. 1:**
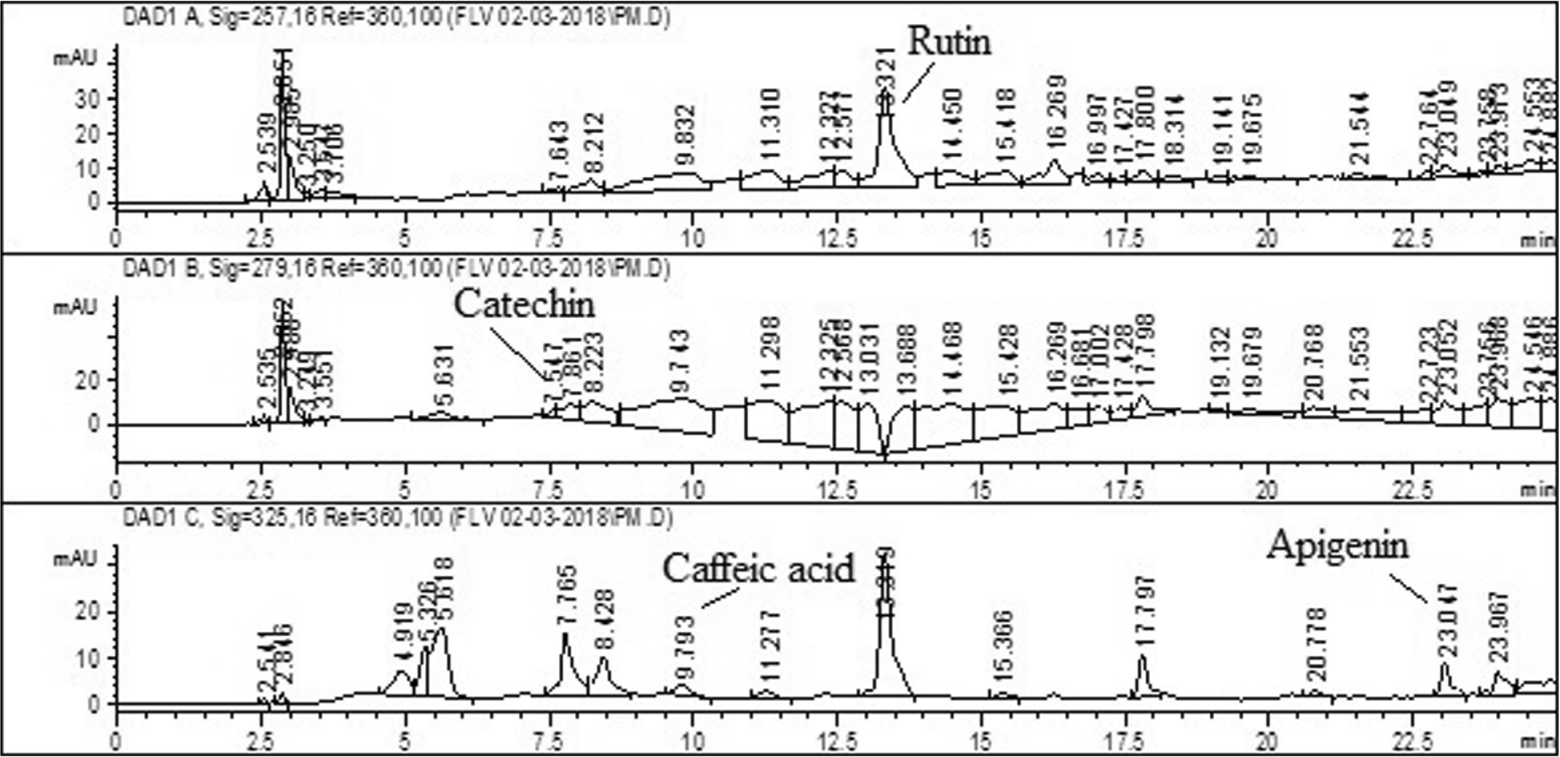
HPLC analysis of PUM at 257, 279 and 325 nm

Eight analytes were subjected to HPLC-DAD method. PUM prototype compounds were identified based on the comparison of retention time and absorption spectra with those of standard (Fig. 1). HPLC spectrogram indicated that among 8 standards 4 compounds were verified as rutin (6.68 ± 0.04 μg/mg), catechin (1.95 ± 0.03 μg/mg), apigenin (0.302 ± 0.01 μg/mg) and caffeic acid (3.94 ± 0.015 μg/mg) in PUM. Result also showed that rutin was the most abundant compound, followed by caffeic acid and catechin, while apigenin was the least abundant compound.

### Acute toxicity and hematological profile

PUM (4% obtained yield) wan administered at a dose of (150 mg/kg and 300 mg/kg bw) disclosed no obvious symptoms of toxicity and mortality in rats. The tested doses did not show any weight loss, physical or clinical changes in female rats. However, after being treated with single dose, plant extract significantly (*P* < 0.05) elevated (~ 2 folds) white blood cells (WBCs) at 1500 mg/kg bw and 3000 mg/kg bw treated rats (Table 2). Red blood cells (RBCs) showed non-significant differences (*P* > 0.05) among treated and control rats. However, hemoglobin (g/dl) level significantly (*P* < 0.05) increased at higher dose. Similarly, lymphocytes and mean corpuscular hemoglobin concentration (MCHC) showed substantial increase (~ 2–3 folds) in treated rats, whereas neutrophil count revealed non-significant differences (*P* > 0.05) among treated and control group.

**Table 2:** Effect of different concentrations of PUM on hematological parameters

Treatment groups	WBC (10^3^/mm^3^)	RBC (10^6^/mm^3^)	HGB (g/dl)	MCV fL	PLT (105/mm3)	LYM (10^3^/mm^3^)	Neutrophil (10^3^/mm^3^)	MCHC (g/dl)	HCT (%)
Control	6.90 ± 0.49^c^	5.93 ± 0.56^a^	12.76 ± 0.36^b^	77.71 ± 1.14^b^	260.30 ± 0.61^c^	3.33 ± 0.60^b^	34.50 ± 0.65^a^	4.17 ± 0.68^b^	40 ± 0.81^c^
PUM (1500 mg/kg bw)	12.70 ± 0.16^**a**^	6.27 ± 0.12^a^	13.20 ± 0.53 ^ab^	35.67 ± 0.20^c^	291.30 ± 0.88^b^	9.77 ± 0.38^a^	38.17 ± 3.40^a^	11.17 ± 0.36^a^	58.13 ± 0.83^a^
PUM (3000 mg/kg bw)	11.26 ± 0.50^b^	6.53 ± 0.29^a^	14.10 ± 0.21^a^	85.40 ± 0.94^a^	321.31 ± 0.88^a^	8.33 ± 0.60^a^	37.17 ± 0.61^a^	9.80 ± 0.63^a^	49.16 ± 0.62^b^

Mean ± SD (*n* = 5 female rats); Means not sharing common superscript (^a-c^) indicate significance difference at *P* > 0.05. PUM; methanol extract of *Pilea umbrosa*, *MCV* Mean corpuscular volume, *MCHC* Mean corpuscular hemoglobin concentration, *MCV* Mean corpuscular volume, *TLC* Total leukocyte count, *PLT* Platelet count, *HGB* Hemoglobin, *WBC* White blood cells, *RBC* Red blood cells, *LYM* Lymphocytes, *HCT* Hematocrit

### Organ and body weight analysis

The effect of PUM on body weight as well as on absolute and relative weight of liver was determined and presented in Table 3. Upon treatment with CCl_4_, significant (*P* < 0.05) increase in the absolute and relative weight of liver was observed while with a notable decrease in body weight. By contrast, simultaneous administration of silymarin and CCl_4_ restored the liver and body weight towards the control rats. The administration of PUM (150 mg/kg and 300 mg/kg bw) in CCl_4_ treated rats significantly (*P* < 0.05) restored the body weight towards the control rats. In addition, co-administration of PUM normalized the absolute and relative weight of liver as compared to CCl_4_ treated group. Likewise, all rats treated with only PUM dose of 150 mg/kg bw and 300 mg/kg bw manifested a significant (*P* < 0.05) increment in body weight when compared with the control group.

**Table 3:** Effects of PUM on liver and body weight of rat

Treatment groups	Initial body weight (g)	Final body weight (g))	% increase in body weight	Absolute liver weight (g)	Relative liver weight (mg/g of body weight)
Control	122.70 ± 2.05	219.33 ± 3.39	79.08 ± 0.52^c^	8.16 ± 0.23^cd^	37.18 ± 0.78^cd^
CCl_4_ (1 ml/kg bw)	126.33 ± 2.62	177 ± 3.56	40.10 ± 0.79^f^	9.12 ± 0.08^a^	51.56 ± 1.02^a^
CCl_4_ + Silymarin (200 mg/kg bw)	119.30 ± 0.94	187.67 ± 2.05	57.25 ± 0.76^e^	8.37 ± 0.09^bc^	44.59 ± 0.97^b^
CCl_4_ + PUM (150 mg/kg bw)	121.70 ± 2.36	193 ± 3.55	58.63 ± 0.38^e^	8.70 ± 0.08^ab^	45.09 ± 0.97^b^
CCl_4_ + PUM (300 mg/kg bw)	123.25 ± 2.05	215.25 ± 3.26	74.64 ± 0.25^d^	8.40 ± 0.12^bc^	39.02 ± 0.57^c^
PUM (150 mg/kg bw)	122.50 ± 2.50	224 ± 4	82.86 ± 0.46^b^	8.38 ± 0.14^bc^	37.39 ± 0.65^c^
PUM (300 mg/kg bw)	120 ± 0	226.50 ± 1.11	88.75 ± 0.93^a^	7.90 ± 0.18^d^	34.87 ± 0.82^d^

Mean ± SD (*n* = 6 male rats); Means not sharing common superscript (^a-f^) indicate significance difference at *P* > 0.05. *PUM* Methanol extract of *Pilea umbrosa*

### Serum analysis of rat

Compared to control group, treatment with CCl_4_ significantly (*P* < 0.05) increased the level of serum enzymes; ALP, ALT, AST and total bilirubin (Table 4). On the other hand, a notable decline in albumin concentration was observed with CCl_4_ administration to rat. Treatment with silymarin (200 mg/kg bw) significantly (*P <* 0.05) inhibited the CCl_4_ elevated level of serum markers, while raising the concentration of albumin protein. As expected, simultaneous administration of either low or high dose of PUM (150 mg/kg, 300 mg/kg bw) with CCl_4_ had shown a pronounced effect (*P <* 0.05) in decreasing the level of these serum enzymes, yet an increase in albumin level was also observed.

**Table 4:** Effect of PUM on serum biochemical markers of liver

	ALT (u/l)	AST (u/l)	ALP (u/l)	Albumin (mg/dl)	Bilirubin (mg/dl)
Control	37.80 ± 1.93^e^	68.46 ± 0.75^e^	60.20 ± 3.02^e^	4.61 ± 0.16^a^	0.59 ± 0.03^e^
CCl_4_ (1 ml/kg bw)	125.70 ± 1.57^a^	147.30 ± 0.74^a^	168.40 ± 2.42^a^	2.27 ± 0.14^d^	1.71 ± 0.04^a^
CCl_4_ + Silymarin (200 mg/kg bw)	47.33 ± 1.24^d^	76.76 ± 0.89^cd^	85.40 ± 1.82^c^	3.80 ± 0.16^bc^	0.79 ± 0.05^cd^
CCl_4_ + PUM (150 mg/kg bw)	89.67 ± 0.94^b^	122.66 ± 1.24^b^	95.76 ± 1.35^b^	2.84 ± 0.07^d^	1.04 ± 0.08^b^
CCl_4_ + PUM (300 mg/kg bw)	52.17 ± 0.84^c^	80.36 ± 2.08^c^	88.56 ± 2.15^bc^	3.61 ± 0.06^c^	0.84 ± 0.04^c^
PUM (150 mg/kg bw)	46.47 ± 1.30^d^	72.46 ± 0.33^de^	75.20 ± 2.33^d^	4.26 ± 0.26^ab^	0.71 ± 0.02^cde^
PUM (300 mg/kg bw)	32.77 ± 1.15^f^	74.53 ± 1.90^d^	65.11 ± 0.99^e^	4.44 ± 0.21^a^	0.63 ± 0.06^de^

Mean ± SD (*n* = 6 male rats); Means not sharing common superscript (^a-f^) indicate significance difference at *P* > 0.05. *PUM* Methanol extract of *Pilea umbrosa, AST* Aspartate transaminase, *ALT* Alanine transaminase, *ALP* Alkaline phosphatase

### Antioxidant status in PUM-treated rats

**Fig. 2:**
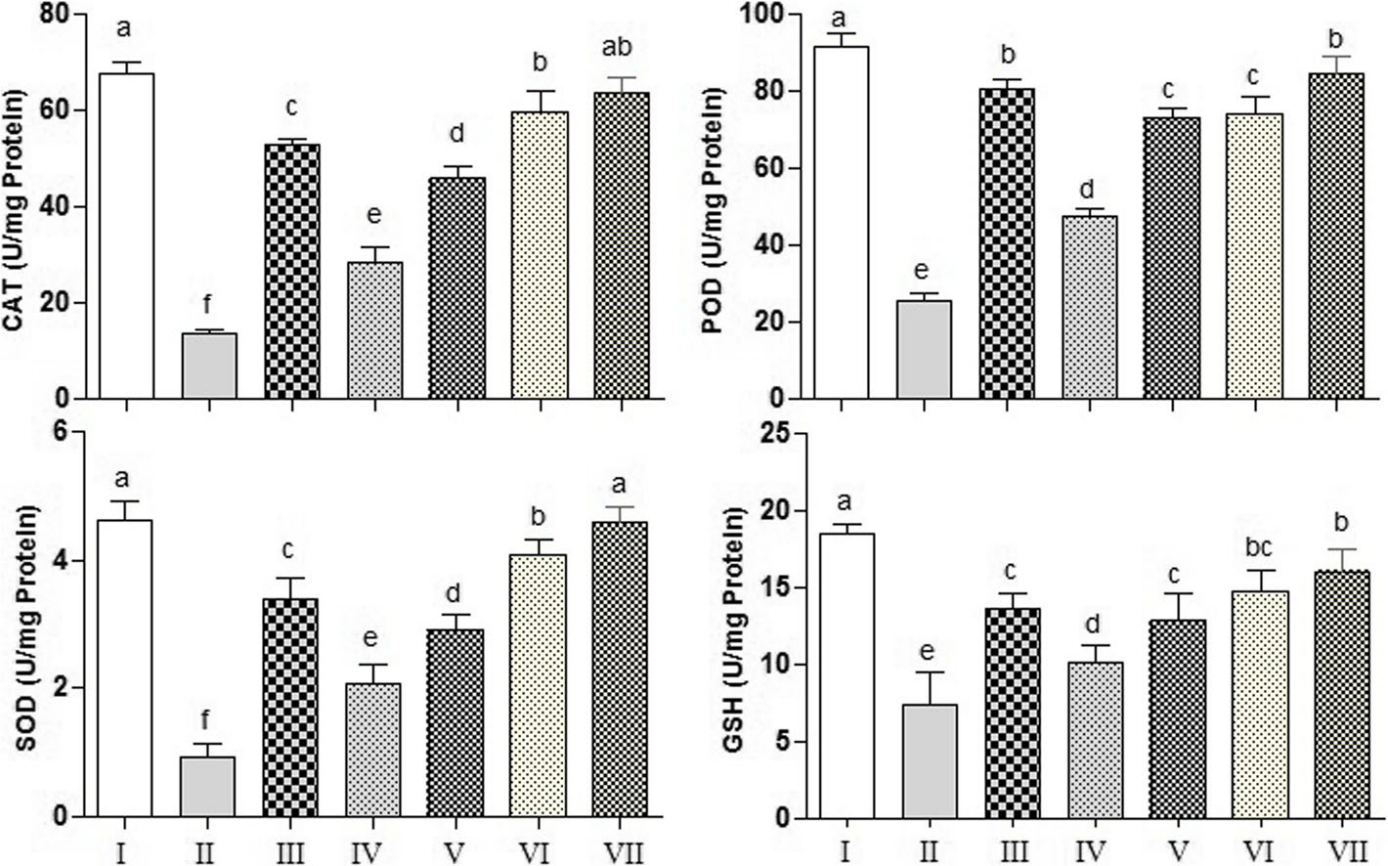
Effect of PUM on antioxidant enzymes in rat liver. CAT; catalase activity, POD; peroxidase activity, SOD; superoxide dismutase activity, GSH; glutathione content. I; Control group, II; CCl_4_ (1 ml/ kg bw) treated group, III; CCl_4_ + Silymarin (200 mg/kg bw) treated group, IV; CCl_4_ + PUM (150 mg/kg bw) treated group, V; CCl_4_ + PUM (300 mg/kg bw) treated group, VI; PUM (150 mg/kg bw) treated group, VII; PUM (300 mg/ kg bw) treated group. Mean ± SD (*n* = 6). Bars with different alphabet letter indicate significance with each other at *P* < 0.05

Investigation on antioxidant status in the liver of treated rats is illustrated in (Fig. 2a-d). In CCl_4_ treated group, we noticed a tissue specific damage accompanied by significant (*P* < 0.05) decline in level of antioxidant enzymes such as CAT, SOD, POD and GSH. However, rats administered with standard drug silymarin along with CCl_4_ protected liver injury, as evidenced by significantly (*P* < 0.05) increased level of these antioxidants. Exposure with PUM at a dose of 150 mg/kg/300 mg/kg combined with CCl_4_, significantly (*P <* 0.05) raised their level towards the control rats. However, administration with either low or high dose of PUM (150 mg/kg bw, 300 mg/kg bw) exhibited slightly lower activity of these enzymes from control group.

### Biochemical indices of liver in PUM-treated rats

From the Table 5, we found significant (*P <* 0.05) increase in the level of biochemical indices such as total protein, H_2_O_2_, nitrite and TBARS in the liver of CCl_4_-treated groups except the level of total protein. Administration of silymarin 200 mg/kg demonstrated a significant (*P <* 0.05) restoration in these biochemical markers by relieving hepatotoxicity induced by CCl_4_. A similar restoration trend was noticed when exposed with PUM at a dose of 300 mg/kg bw as compared to low dose (150 mg/kg bw), suggesting nearly similar effect of PUM to silymarin at maximum dose. Moreover, exposure to either dose of PUM alone did not affect the levels of examined biochemical markers across treated groups.

**Table 5:** Effects of PUM on hepatic biochemical markers of rat

	Protein (μg mg − 1 tissue)	TBARS (nM mg − 1 protein)	Nitrite (nM ml − 1)	H_2_O_2_ (nM mg − 1 protein)
Control	15.55 ± 0.32^a^	36.41 ± 4.58^d^	44.40 ± 1.79^d^	4.23 ± 0.44^e^
CCl_4_ (1 ml/kg bw)	11.09 ± 0.33^f^	67.16 ± 4.74^a^	66.17 ± 1.72^a^	9.29 ± 1.22^a^
CCl_4_ + Silymarin (200 mg/kg bw)	14.37 ± 0.22^c^	46.43 ± 2.54^c^	44.85 ± 2.44^cd^	5.62 ± 0.20^d^
CCl_4_ + PUM (150 mg/kg bw)	13.37 ± 0.34^e^	52.80 ± 3.35^b^	54.77 ± 6.19^b^	8.15 ± 0.05^b^
CCl_4_ + PUM (300 mg/kg bw)	14.18 ± 0.35^d^	49.54 ± 2.55^b^	52.86 ± 2.59^bc^	6.65 ± 0.32^c^
PUM (150 mg/kg bw)	14.88 ± 0.36^b^	38.72 ± 5.21^d^	42.18 ± 0.96^d^	5.94 ± 0.37^cd^
PUM (300 mg/kg bw)	15.27 ± 0.29^ab^	41.01 ± 4.00^d^	44.85 ± 1.35^cd^	5.35 ± 0.65^d^

Mean ± SD (*n* = 6 male rats); Means not sharing common superscript (^a-f^) indicate significance difference at *P* > 0.05. *PUM* Methanol extract of *Pilea umbrosa, TBARS* Thiobarbituric acid reactive substances

### Effect of PUM extract on CCl_4_-induced hepatic ER stress

**Fig. 3:**
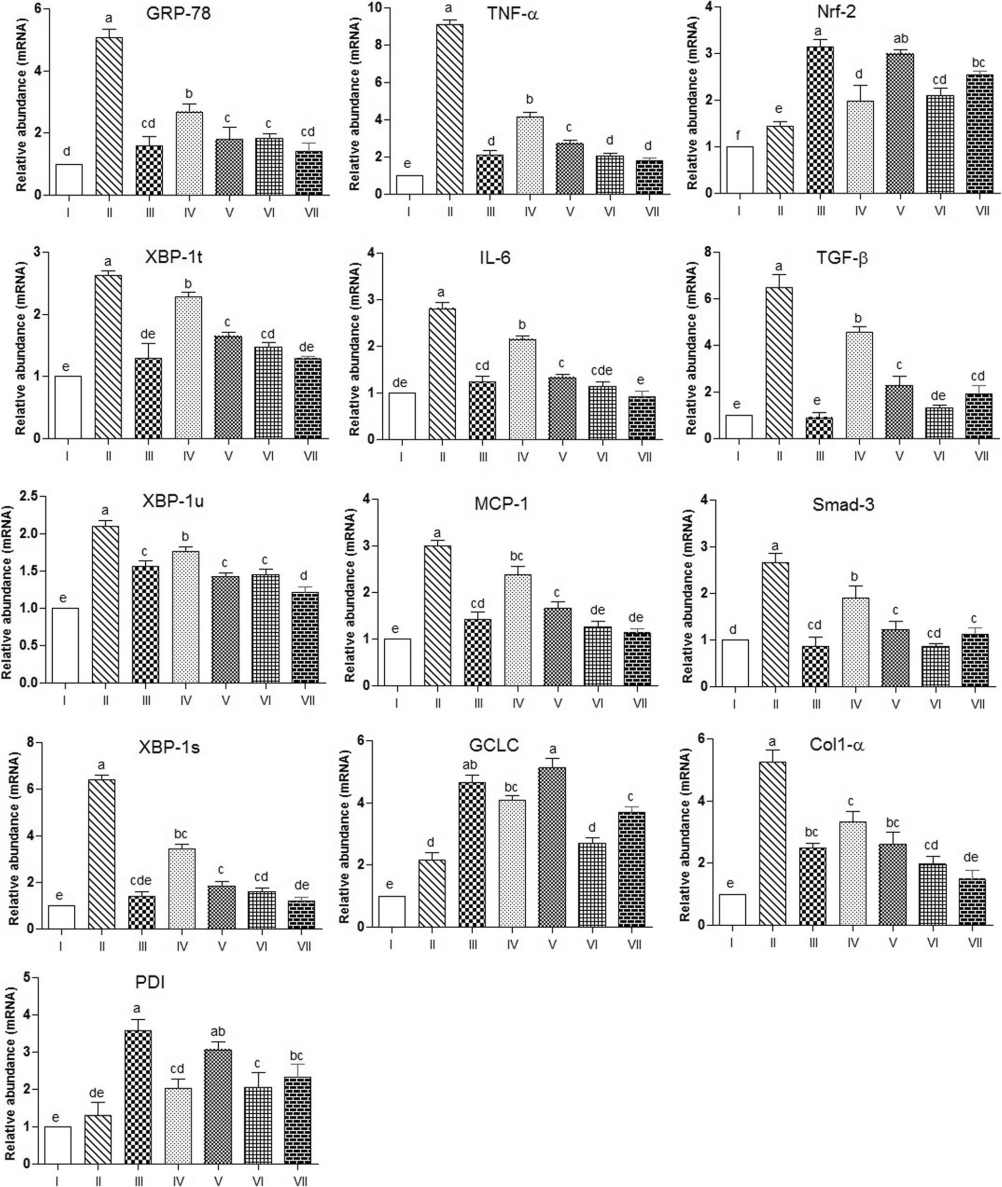
Graphical representation of different treatments of PUM on mRNA expressions of various genes involved in multiple pathways i.e., ER stress, inflammation and fibrosis. I; Control group, II; CCl_4_ (1 ml/ kg bw) treated group, III; CCl_4_ + Silymarin (200 mg/kg bw) treated group, IV; CCl_4_ + PUM (150 mg/kg bw) treated group, V; CCl_4_ + PUM (300 mg/kg bw) treated group, VI; PUM (150 mg/kg bw) treated group, VII; PUM (300 mg/ kg bw) treated group. PUM; *Pilea umbrosa* methanol extract. Mean ± SD (*n* = 3). Bars with different alphabet letter indicate significance with each other at *P* < 0.05

To explore the effect of PUM on CCl_4_-mediated hepatic ER stress, we detected the mRNA expression of hepatic ER stress related proteins. As revealed in (Fig. 3a-e), the mRNA level of GRP78, XBP-t, XBP1-u and XBP1-s PDI was decreased significantly (*P* < 0.05) in CCl_4_ intoxicated group compared with the control group. However, after treatment with silymarin along with CCl_4_, a significant (*P* < 0.05) increase was obtained in the level of ER stress related protein mRNA expressions. However, co-administration of PUM extract 300 mg/kg with CCl_4_ had shown considerably significant (*P* < 0.05) effect in alleviating the ER stress related protein mRNA expression. Furthermore, non-significant differences were found among both PUM doses tested at 150 mg/kg and 300 mg/kg bw on ER stress markers when compared with control or each other. These results indicated that PUM significantly (*P* < 0.05) decreased the level of ER stress markers upregulated in UPR pathway.

### Effect of PUM on CCl_4_-induced hepatic inflammation and antioxidant enzymes

As shown in (Fig. 3f-h), the expression of hepatic inflammatory markers including cytokines TNF-α, IL-6 and chemokine MCP-1 in treated and control groups were determined. CCl_4_ treatment significantly (*P* < 0.05) up-regulated the mRNA level of TNF-α, IL-6 and MCP-1,when compared with the control group. Treatment with silymarin at 200 mg/kg bw along with CCl_4_ had significantly (*P* < 0.05) down regulated the mRNA level of these markers relative to the CCl_4_ group. Similarly, co-administration of PUM (300 mg/kg) with CCl_4_ had remarkably diminished the mRNA level of TNF-α, IL-6 and MCP-1 thereby reducing hepatic inflammation. Similarly, PUM treatment alone had not shown any significant effect (*P* > 0.05) in altering the level of these markers relative to the control group. In addition, the mRNA expression level of antioxidant enzymes GCLC and Nrf-2 was significantly (*P* < 0.05) reduced after CCl_4_ intoxication (Fig. 3). However, the effect of PUM at 300 mg/kg was found effective in increasing the expression of antioxidant enzymes regarding to CCl_4_ treated group.

### Effect of PUM extract on CCl_4_-induced hepatic fibrosis

In order to investigate the effect of PUM on hepatic fibrosis, we detected the hepatic mRNA expressions of TGF-β, Smad-3 and Col1-α. As presented in (Fig. 3), the mRNA expression level of TGF-β, Smad-3 and Col1-α in CCl_4_ intoxicated group were significantly aggregated (*P* < 0.05) relevant to the control group. Simultaneous administration of silymarin at 200 mg/kg bw with CCl_4_ significantly reduced the expression level of these markers. Interestingly, PUM co-treatment at 300 mg/kg with CCl_4_ also significantly reduced the mRNA expression of these genes. It was observed that rats treated solely with PUM extract (150 mg/kg bw, 300 mg/ kg bw) had shown non-significant decrease on mRNA expression levels of fibrosis markers from control group indicating the hepatoprotective effect of PUM extract.

### Histopathological analysis

**Fig. 4:**
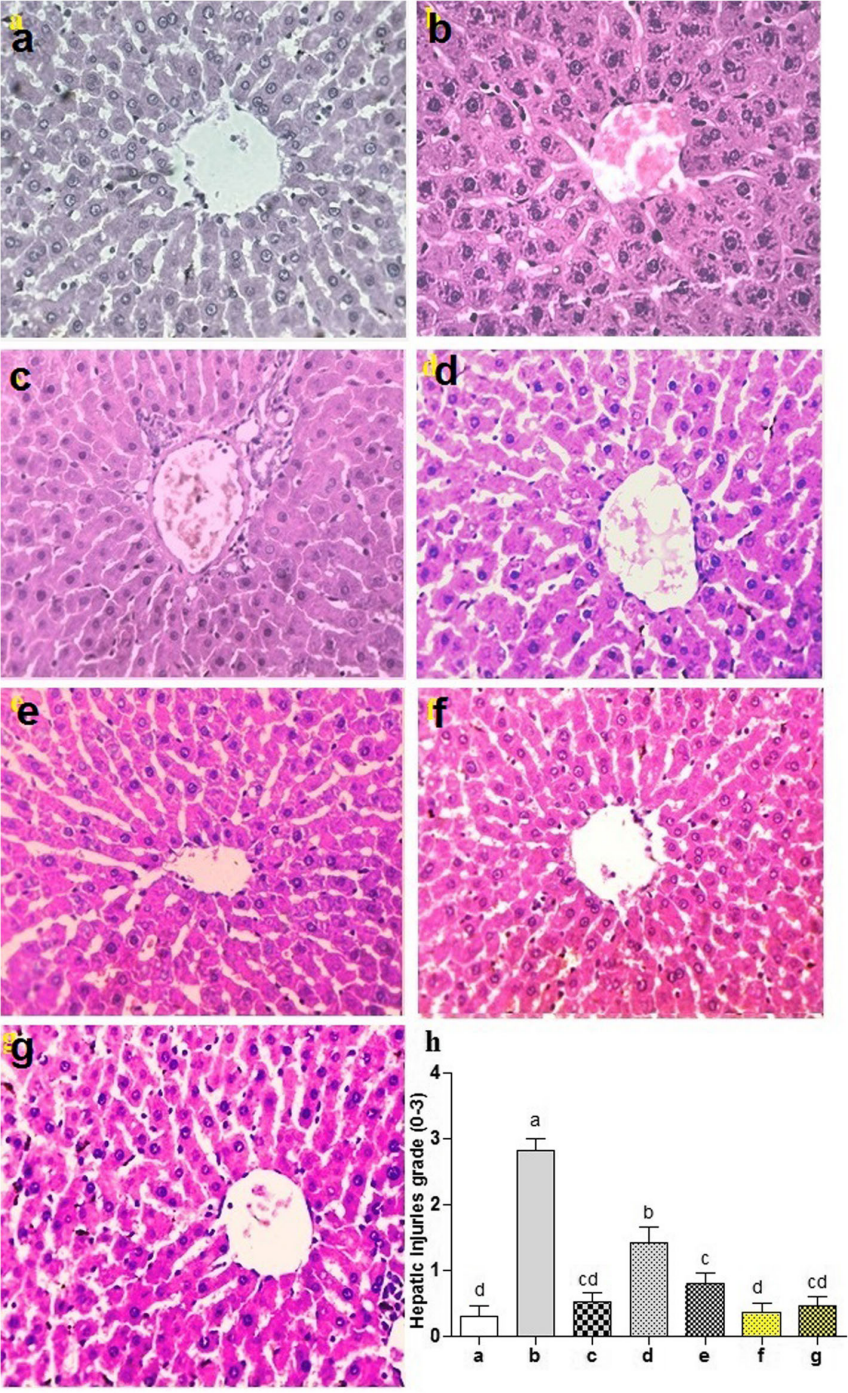
Protective potential of PUM on liver histopathology of rats (40× magnification with hematoxylin-eosin stain). **a**; Control group, **b**; CCl_4_ (1 ml/kg bw) treated group, **c**; CCl_4_ + Silymarin (200 mg/kg bw) treated group, **d**; CCl_4_ + PUM (150 mg/kg bw) treated group, **e**; CCl_4_ + PUM (300 mg/kg bw) treated group, **f**; PUM (150 mg/kg bw) treated group, **g**; PUM (300 mg/kg bw) treated group, **h**; hepatic injuries ranges from 0 to 3. PUM; *Pilea umbrosa* methanol extract. Mean ± SD (*n* = 6). Bars with different alphabet letter indicate significance with each other at *P* < 0.05

Histopathological examination indicated normal liver morphology in control group (Fig. 4a). Liver section treated with CCl_4_ showed severe injuries including focal area of severe perivascular and lobular necrosis with neutrophil infiltration, cellular hypertrophy and sinusoidal obstruction (Fig. 4b). Co-treatment with silymarin recovered liver morphology and showed slight perivascular necrosis along with clear and distinct hepatocyte appearance (Fig. 4c). However, at 150 mg/kg bw of PUM extract, few non-patterned areas with mild necrosis, less neutrophil infiltration and steatosis were seen, but there were no extensive changes found on histological features of liver section treated with 300 mg/kg bw in CCl_4_ treated group (Fig. 4d & e). Treatment with PUM alone at either low or high dose had shown quite normal histological features as in control group (Fig. 4f & g). Thus, histological examination indicated the hepatoprotective effect of PUM with less necrosis and leukocyte infiltration.

### Studies on markers of liver injuries

Different liver injuries obtained after CCl_4_ administration to rat and the ameliorative potential of PUM is shown in Table 6. Liver injuries such as steatosis, leukocyte infiltration, fatty degeneration, perivascular and lobular necrosis, cellular hypertrophy and sinusoidal obstruction were recorded in very severe range after CCl_4_ treatment to rat. Amelioration of CCl_4_-induced liver injuries in rat was recorded in a lower to medium range with co-treatment of silymarin to rat. On the other hand medium to severe grade and lower to medium grade liver injuries were recorded with co-administration of PUM (150 mg/kg bw and 300 mg/kg bw), respectively. However, different liver injuries in PUM (150 mg/kg bw) and PUM (300 mg/kg bw) alone treated rats were recorded in very minor grade or in lower grade.

**Table 6:** Assessment of markers of liver injuries in rat

Treatment groups	Liver injuries					
	Steatosis	Leukocyte infiltration	Fatty degeneration	Perivascular & lobular necrosis	Cellular hypertrophy	Sinusoidal obstruction
Control	+/−	+/−	+/−	+/−	+/−	+/−
CCl_4_ (1 ml/kg bw)	+++	+++	+++	+++	+++	+++
CCl_4_ + Silymarin (200 mg/kg bw)	+	+	++/−	+	+	+
CCl_4_ + PUM (150 mg/kg bw)	++/−	++	++	++/−	++	++/−
CCl_4_ + PUM (300 mg/kg bw)	+	++/−	++/−	+	++/−	+
PUM (150 mg/kg bw)	+/−	+/−	+/−	+/−	+/−	+/−
PUM (300 mg/kg bw)	+/−	+/−	+	+/−	+	+/−

+/−; very minor, +; lower, ++/−; medium, ++; severe, +++; very severe. *PUM* Methanol extract of *Pilea umbrosa*

## Discussion

Exposure to xenobiotics provokes liver dysfunction which subsequently leads to inflammation, ER stress and induces fibrosis. In order to overcome these detrimental effects, certain natural antioxidants having scavenging activity have been reported recently showing beneficial effects on the human body [30, 31]. Plants rich in polyphenols and flavonoids exhibit a wide range of biological effects such as antioxidant, hepatoprotective, anticancer, anti-diabetic and anti-inflammation [32]. In this regard, we have analyzed the hepatoprotective effects of PUM plant against CCl_4_–induced damaging effects on the rat liver. HPLC analysis confirmed the presence of phytoconstituents in an appreciable amount, such as polyphenols and flavonoids compounds in PUM including rutin, catechin, caffeic acid and apigenin. These secondary metabolites have been reported in varieties of medicinal properties. Rutin revealed admirable hepatoprotective, antiplatelet, anti-inflammatory and antioxidant activities [33]. Catechin and caffeic acid showed protection against neurological disorders, inflammation and apoptosis due to anti-oxidant potential whereas for apigenin anti-inflammatory property was reported [34]. The presence of these secondary metabolites in admiring amount in PUM revealed its potential anti-oxidant and hepatoprotective effect.

The present study declared that based on hematological parameters, PUM is considered as non-toxic when examined on rat model and had not posed an adverse effect on count of WBC, RBC, MCV, HB, HCT and platelets at maximum dose of 3000 mg/kg. Moreover, mortality and any obvious symptoms of abnormal physical behavior also had not been observed. Hence, these findings are supported by the previous studies which were performed with methanol extract of other plant species [35].

Screening of liver and relative body weight serve as an indicator for the general health status of animals [36]. Data indicated that the group treated with CCl_4_ led to an increase in liver weight and decrease in ratio of liver/body weight compared to control. On the contrary, treatment with PUM along with CCl_4_ showed an increase in liver and body weight near to control in a dose dependent manner. Initially, we observed weight loss in rats exposed with CCl_4_ but after treatment with PUM both liver and body weight of rat was restored to normal.

Upon biotransformation, CCl_4_ converts into free radicals and causes hepatic injury or leakage in cell membrane which results in the discharge of liver enzymes into the blood stream. The serum level of AST, ALT, ALP, bilirubin and albumin that are used as marker for screening of liver function [37] were altered after treatment with CCl_4_ as compared to control. Whereas treatment with PUM to CCl_4_ intoxicated rats had dose dependently restored their level towards the control group. Therefore, these results confirmed that the PUM has significant preventive potential for hepatic injuries.

Antioxidant enzymes; CAT, POD and SOD or a molecule such as glutathione in liver tissue play important role against reactive oxygen species and show curative activity against liver inflammation [38]. These biochemical markers have the ability to scavenge superoxide anion and hydrogen peroxide thereby reduces their toxic effects. SOD protects hepatic cells by dismutation of superoxide radical into hydrogen peroxide and oxygen [39]. CAT neutralizes the toxicity of H_2_O_2_ and decomposes it into water and oxygen. Glutathione forms a disulfide bond with H_2_O_2_ and decreases its concentration. Altogether, these serum markers are associated with the first line of defense against free radical mediated stress [40]. In CCl_4_ treated group, we detected a drastic decrease in the level of these antioxidant enzymes which corresponds to disease severity. On the other hand, CCl_4_ generated free radicals might also be involved in inactivation of antioxidant enzymes [41]. In contrast, the group administered with PUM prior to CCl_4_ treatment revealed significant dramatic increase in antioxidant enzymes parallel to the amount raised in the silymarin treated group. We could demonstrate that these results confirm the significant effect of PUM in the protection of liver by increasing antioxidant enzymes to minimize inflammation. These results were in line with our serum markers findings.

Generally, CCl_4_ delivers chloride (Cl־) ion that reacts with poly unsaturated fatty acids and causes their peroxidation which eventually causes deformity in hepatocyte cell membrane. This shift generates highly reactive aldehydes (TBARS) an indicator of oxidative injuries, that later on triggers accumulation of ECM and fibroblast proliferation [42, 43]. Radicals of nitric oxide and H_2_O_2_ also damage HSCs and Kupffer cells implying lipid peroxidation thereby facilitating inflammation and fibrogenesis [44]. In the present study, except protein concentration the level of nitrite radical, TBARS and H_2_O_2_ were elevated in rat group challenged with CCl_4_. However, PUM treatment in combination with CCl_4_ effectively restored the level of these liver biochemical markers closer towards the control rats, thus prevented lipid peroxidation [45]. Therefore, based on these findings we can suggest that PUM has anti-inflammatory activity which is most likely corresponds to its high content of polyphenol and flavonoid such as rutin, caffeic acid and catechin. These findings are consistent with previously published study [46]. In addition, a growing body of reports have been emphasizing the hepatoprotective mechanism of various genera from CCl_4_ induced lipid peroxidation and cell damage [47].

In response to stress mediated by ROS, homeostasis of ER becomes dysfunctional, which triggers accumulation of unfolded proteins due to which ER prompts a protective response named as unfolded-protein response (UPR) intended to restore homeostasis. Recent studies have demonstrated that upon ER stress, UPR through its IRE1-α branch elicits cleavage of XBP-1, generating XBP-1 s transcription factor that eventually associated with ERAD and transcription of chaperones (associated with protein folding) such as protein disulfide isomerase (PDI). In prolonged stress if the damage is not repairable, ER provoked inflammatory and cell death pathways through activation of caspases [48, 49]. As evidenced by our previous study, methanol extract of PUM effectively enhanced liver protection activity with activation of GRP-78 and XBP-1 s of UPR1 pathway at molecular level. Therefore, we attempted to reveal the effect of PUM against ER stress. Findings indicated that consistent with above mentioned mechanism, CCl_4_ causes down regulation of GRP-78 and XBP-1 s and PDI, hence indicated ER stress, whereas upregulation of these markers after applying PUM confirmed the hepatoprotective activity of this plant via activation of IRE1-α branch of UPR pathway (Fig. 3a-e). Moreover stimulation of PERK branch of UPR phosphorylate nuclear erythroid 2 p45-related factor 2 (Nrf-2), a transcription factor for antioxidant enzymes such as glutathione-S-transferase has been implicated in this study [50]. In addition, an antioxidant enzyme regulator γ-glutamylcysteine ligase (GCLC) considered as a centralized enzyme regulator for glutathione synthesis has also been observed. Therefore, in CCl_4_ intoxicated group the level of Nrf-2 and GCLC were remarkably reduced when compared with the control group. However, PUM treatment reciprocated their level towards the control rat and suggesting that protective effect of PUM is critical because of its increased antioxidant enzyme level.

Besides, UPR has also been associated with release of pro-inflammatory cytokines through IRE1-α mediated activation of nuclear factor kappa B (NF-кB) and c-jun N-terminal kinase (JNK) pathways. In addition upon chronic liver injury, Kupffer cells excessively release pro-inflammatory cytokines (IL-6 and TNF-α) that subsequently activate HSCs to produce MCP-1 (chemokine) which aggregate inflammation [31]. Upon CCl_4_ treatment, we observed an increase in the level of IL-6, TNF-α and MCP-1. Treatment with PUM has tremendous effect to forestall the level of these inflammatory mediators. These results confirmed the anti-inflammatory activity of PUM which is consistent with our previous finding [10].

Next, we attempted to analyze the role of PUM in evading hepatic fibrosis. TGF-β/Smad signaling pathway causes liver fibrosis by hyper activation of TGF-β, Smad-3 and Col1-α [51]. A previous report showed inhibition of fibrosis by deactivation of HSCs over TGF-β/Smad interference [14]. These results indicated that PUM extract showed anti-fibrotic potential by suppressing the mRNA expression of TGF-β, Smad-3 and Col1-α. Current study hence revealed that PUM is considerably resilient to fibrosis by inhibiting TGB-β/Smad pathway.

Histopathology examination of liver revealed the non-toxic property of PUM in control group depicting normal hepatocytes architecture whereas the photomicrograph of CCl_4_ intoxicated group showed liver injury with necrosis, massive fibrosis, neutrophils infiltration and vacuolar degeneration. The high dose of PUM (300 mg/kg) and silymarin treatment depicted symmetrical arrangement of liver sinusoids along with normal hepatocytes morphology. Furthermore, we observed decrease in inflammation and reduction in dilation of central vein with normal cellular morphology which might be due to rapid regenerative property of PUM in a dose dependent manner (Karp, 2009). This finding revealed the nearly similar efficacy of high dose of PUM to silymarin standard drug. In addition, the non-toxic nature was also confirmed for PUM by microscopic examination of the group treated with PUM solely. This examination also illustrated that PUM does not cause hypertrophy or alteration in cell structure.

## Conclusion

Taken together, our study illustrated the existence of a high amount of polyphenolic compounds in PUM. Our studies confirmed the anti-inflammatory, anti-fibrosis and hepatoprotective activity of PUM at biochemical and molecular level, which could be explained on the basis of an appreciable amount of flavonoids, phenolics and other antioxidants present in PUM extract. Therefore, we conclude that administration of defined concentration of PUM extract can reduce liver ailments. Future studies on PUM extract at low to medium concentration (150 mg/kg bw, 300 mg/kg bw) to be used as potential therapeutic agent for liver diseases and isolation of active compounds involved in the above mentioned activities are highly needed.

## Data Availability

The data may be available on request.

## Abbreviations

PUM: Methanol extract of *Pilea umbrosa*
AST: Aspartate transaminase
ALT: Alanine transaminase
ALP: Alkaline phosphatase
MCV: Mean corpuscular volume
MCHC: Mean corpuscular hemoglobin concentration
MCH: Mean corpuscular hemoglobin test
TLC: Total leukocyte count
PLT: Platelet count
ER: Endoplasmic reticulum
(GRP-78): Glucose regulated protein
XBP-1 t: x-box binding protein1-total
XBP-1 u: x-box binding protein1-unspliced
XBP-1 s: x-box binding protein1-spliced
TNF-α: Tumor necrosis factor-α
IL-6: Interleukin-6
MCP-1: Monocyte chemoattractant protein-1
GCLC: γ-glutamylcysteine ligase
PDI: Protein disulfide isomerase
Nrf-2: Nuclear erythroid 2 p45-related factor 2
TGF-β: Transforming growth factor-β
Col1-α: Collagen type 1
